# Pituitary Adenoma with Calcifications: A Case Report

**DOI:** 10.7759/cureus.5542

**Published:** 2019-08-31

**Authors:** Yakoot Khan, Noor Malik, Saba I Awan, Syed Hassan Khalid, Altaf Ali Laghari

**Affiliations:** 1 Neurosurgery, Aga Khan University Hospital, Karachi, PAK; 2 Internal Medicine: Diabetes and Endocrinology, Icahn School of Medicine, New York, USA; 3 Neurosurgery, Aga Khan University Hosiptal, Karachi, PAK

**Keywords:** pituitary adenoma, calcification, ct-scan

## Abstract

Pituitary adenomas and Rathke’s cleft cyst with calcification are rarely seen and craniopharyngioma still remains the common sellar suprasellar space occupying lesion with calcification. Presence of calcification is reported in pituitary adenoma in only 0.2% to 8% cases. The pituitary adenoma with calcification is a rare radiological finding and it must be distinguished from other lesions of the pituitary gland as the management and prognosis differs significantly. We report a case of a 29-year-old gentleman presented electively with the complaints of deterioration of vision for four months. CT-scan without contrast examination revealed pituitary adenoma with calcification. The patient underwent transsphenoidal resection and was discharged on third post-operative day. Histopathology confirmed the diagnosis of pituitary adenoma with calcification. Pituitary tumor presenting with evidence of calcification is an infrequent radiological finding and identification of pituitary adenomas with calcifications is essential as it guides towards medical and surgical management of the lesion.

## Introduction

Pituitary adenoma can atypically present with sellar calcification. Microscopic calcification is seen more commonly than radiological calcification. Calcification in pituitary adenoma is important to recognize as it helps in determining the surgical approach (transsphenoidal vs transcranial). Resection of tumor with calcification can be challenging and other treatment modalities can be considered [1].

## Case presentation

**Figure 1:**
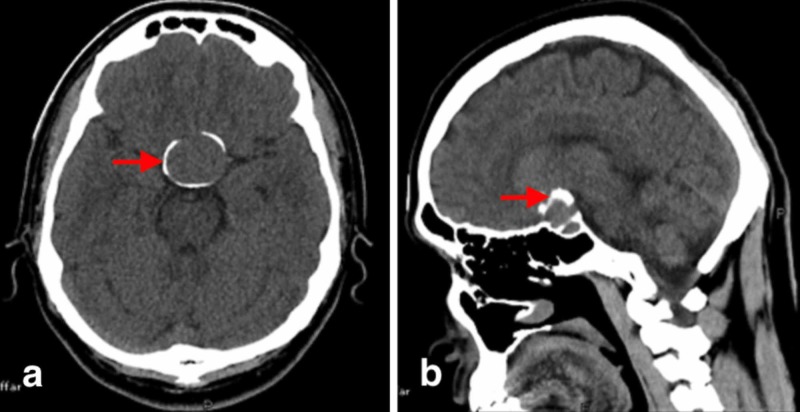
(a) CT scan brain plain, axial section showing round, compact calcification around sellar and suprasellar lesion. (b) CT scan brain sagittal section showing round, compact calcification around sellar and suprasellar lesion.

**Figure 2:**
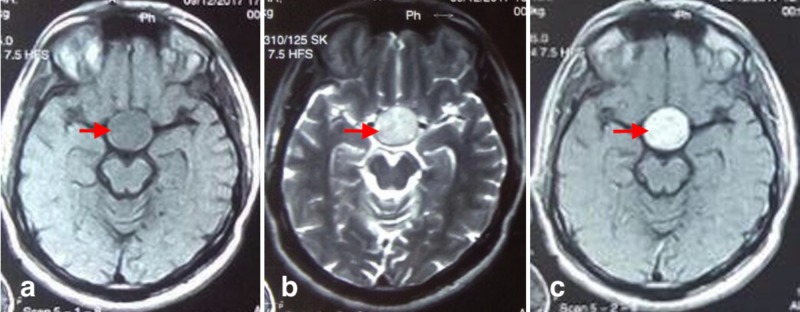
(a) T1-weighted axial image showing hypointense sellar lesion with parasellar extension. (b) T2-weighted axial image showing hyperintense sellar lesion with parasellar extension. (c) T1-weighted image with gadolinium contrast showing diffuse contrast enhancement of the lesion.

A 29-year-old male patient presented to us with the complaint of bilateral temporal visual loss for the last four months. The patient presented electively and had a non-contrast CT scan done to lead to the diagnosis of pituitary adenoma (Figure 1). T1-weighted magnetic resonance imaging (MRI) showed a hypointense suprasellar lesion with parasellar extension. T2-weighted imaging showed a hyperintense lesion and T1-weighted diffuse contrast enhancement of lesion with gadolinium contrast (Figure 2). Endocrinological workup was within the normal range, which suggested that lesion was a non-functioning pituitary adenoma. After pre-op anesthesia assessment, the patient was planned for procedure. Neuronavigation-guided microscopic transsphenoidal resection of pituitary adenoma was performed. Intraoperative finding was soft, suckable, mildly vascular tumor. Intraoperative estimated blood loss was 300 ml. Post-operatively recovery was smooth and uneventful. Visual perimetry showed persistent bilateral temporal hemianopia. The patient was discharged on 3rd post-operative day.

**Figure 3:**
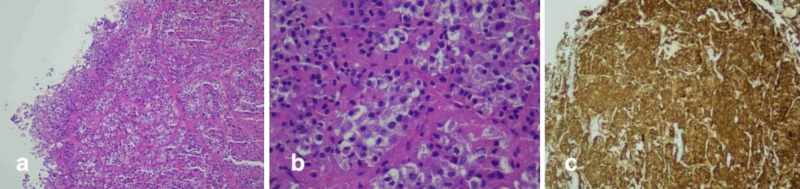
(a & b) Microscopically the tumor exhibits nests (Fig. a →) of monotonous round to oval cells with bland looking central nuclei (Fig. b ←) and granular eosinophilic cytoplasm (Fig. b ↑). Mitotic activity or necrosis is not present. (c) Tumor cells show diffuse strong positive staining for Synaptophysin immunostain.

Histopathology showed multiple grey-brown irregular tissue pieces measuring 2.2 x 1.4 cm in aggregate. Microscopic examination showed fragments of a tumor composed of nests, acini, trabeculae, and cords of monotonous round to oval cells exhibiting bland-looking central nuclei and granular eosinophilic cytoplasm. No mitotic activity was appreciated. No necrosis was seen (Figure 3a, 3b). Immunohistochemical stain Synaptophysin was positive in tumor cells (Figure 3c).

## Discussion

Suprasellar masses most commonly include pituitary adenoma and craniopharyngioma. Craniopharyngioma is a sellar/suprasellar mass that presents with calcification on CT scan which is an almost diagnostic characteristic of the lesion. On radiological evidence, one can differentiate sellar/suprasellar lesion on the basis of various forms of calcifications that include curvilinear, nodular and mixed patterns that can give an idea regarding the nature of the lesion [2]. Patients with craniopharyngioma when compared with pituitary adenoma in majority of the cases show nodular type calcification while presence of curvilinear calcification hints towards pituitary adenoma or Rathke’s cleft cyst [2,3]. Calcification can also be seen in inflammation of the pituitary gland, vasculitis and Rathke’s cleft cyst [2-4].

In patients observed with having cavernous malformations, the vascular lesion most frequently shows calcification, with an incidence rate of 40-60%. These lesions' resemblance is like popcorn kernels [5]. The second observed vascular lesions with calcification are caused by arteriovenous malformations. Aneurysm is much less frequent. The reason for this may be that aneurysms become symptomatic and are treated. That results in less likely outcome of it to become calcified. A well-circumscribed lobe-shaped calcified formation in the suprasellar or parasellar region likely indicates a possible aneurysm. Larger aneurysms tend to calcify, resulting in calcification settling in the walls of the blood vessel, and resulting in intraluminal thrombosis [5].

Meningioma in comparison to a pituitary adenoma is an extra-axial, dural-based mass that on MRI shows hypointense or isointense gray matter on T1-weighted images and hyperintense or isointense on T2-weighted images. There is usually strong, homogeneous contrast enhancement after gadolinium administration. On CT, the meningioma presents as an extra-axial mass that displaces the normal brain. They are smooth in contour, adjacent to dural structures, and sometimes calcified or multilobulated [6].

In our case, the radiological evidence showed sellar suprasellar curvilinear calcification pattern on CT scan that pointed toward pituitary adenoma (Figure 1) and T1-weighted MRI showed a hypointense suprasellar lesion with parasellar extension (Figure 2).

Pituitary adenoma has prevalence of about one in 2688 adults. It presents with clinical features of hypopituitarism, headache and visual defects due to mass effects of a hypo-functioning tumor. Treatment modalities include medical therapies, radiotherapy and surgery [7]. Our patient presented with symptoms of bitemporal hemianopsia for the last 3-4 months.

It has been hypothesized that calcification in pituitary adenomas occurs due dystrophic calcification as a result of progressive tumor enlargement with resultant central ischemic effect. This may also promote osteoid metaplasia in cases where well-differentiated lamellar bone tissue was histologically identified [1,8]. It has also been postulated that calcification occurs as a result of secondary degenerative changes as an outcome of silent pituitary apoplexy [1]. One of the other possibilities is heterotopic induction of calcification secondary to raised intrasellar pressure and compression by the lesion [8]. The hormonal effects of growth hormone (GH), vascular endothelial growth factor, prolactin (PRL) and osteocalcin at the site of the lesion may also lead to calcification [1,9].

The treatment option of pituitary lesions is based on clinical features and imaging as in more than 95% of cases pituitary adenomas can be resected with transsphenoidal approach while transcranial approach remains the mainstay surgical option for craniopharyngioma [2].

The importance of recognition of the presence of calcification and its extent within the lesion may impact the surgical approach. In cases reported in literature, transsphenoidal surgical approach was carried out but in those cases the extent of calcification, tumor size and suprasellar extension played an important role in determining the preferred surgical option [1,10]. Ibrahim et al. presented a case, with a suprasellar region extension with burrowing into medial frontal lobes above planum sphenoidale. The amount of calcification appreciated on pre-operative CT imaging warranted a fronto-temporal craniotomy and a sub-frontal approach because of the tumor size, amount of calcification and suprasellar extension [1]. However, in our case the lesion was extended in the suprasellar region with a curvilinear calcification on pre-operative CT imaging and the tumor was removed transsphenoidally.

## Conclusions

Pituitary tumor with calcification is an infrequent radiological finding and preoperative identification of calcifications is essential as it helps in surgical planning. Here we have reported a patient who presented with a suprasellar pituitary adenoma with parasellar extension and curvilinear calcification which is an uncommon presentation.
